# Effects of movement behaviors on preschoolers’ cognition: a systematic review of randomized controlled trials

**DOI:** 10.1186/s12966-025-01705-y

**Published:** 2025-01-23

**Authors:** Catalina Pacheco, Victoria Culkin, Amelia Putkaradze, Nan Zeng

**Affiliations:** 1 Prevention Research Center, Department of Pediatrics, School of Medicine, University of New Mexico Health Sciences Center, Albuquerque, NM USA; 2 Department of Psychology, University of New Mexico, Albuquerque, NM USA; 3 Department of Chemistry and Biochemistry, Texas Tech University, Lubbock, TX USA; 4 Manning College of Nursing & Health Sciences, Department of Exercise and Health Sciences, University of Massachusetts Boston, Boston, MA USA

**Keywords:** Physical activity, Sedentary behaviors, Sleep, 24-Hour Movement guidelines, Yong Children, Cognitive Development

## Abstract

**Background:**

Movement behaviors, including physical activity (PA), sedentary behavior (SB), and sleep, are fundamental to early childhood development. These behaviors interact dynamically within a 24-hour period, creating a complex balance that influences not only physical health but also cognitive and emotional well-being in young children. While the physical health benefits of movement behaviors are well-documented, systematic evaluations of how interventions targeting these behaviors affect cognitive development in preschool-aged children remain limited.

**Methods:**

This review was guided through PRISMA 2020 guidelines. We conducted a systematic review of randomized controlled trials (RCTs) to evaluate the impact of interventions targeting PA, SB, and sleep on cognitive outcomes in preschool-aged children. A comprehensive search was performed across five databases: PubMed, PsycInfo, Web of Science, Embase, and CINAHL, covering studies published between January 2000 and December 2023. Eligible studies were those that focused on at least one movement behavior, had a minimum intervention duration of four weeks, and assessed cognitive development as a primary outcome. The cognitive outcomes evaluated included executive function, attention, memory, and other key domains critical to early childhood development, such as language, processing speed, and social cognition.

**Results:**

Twenty-two RCTs (14 individual, 8 cluster) met the inclusion criteria. Of these, 21 studies focused on PA, while only one targeted SB, and none specifically addressed sleep or combined movement behaviors. PA interventions, particularly those involving cognitively engaging activities, significantly improved cognitive domains such as executive function, inhibition, and attention, with effect sizes ranging from moderate to large (Cohen’s d > 0.5). The SB-focused study did not report significant cognitive improvements. A clear gap exists in understanding the effects of sleep and multi-behavior interventions on cognitive outcomes.

**Conclusions:**

Cognitively engaging PA interventions demonstrated the largest effects, while motor skill-focused and general PA programs produced moderate to smaller gains. Evidence on SB and sleep interventions remains limited, with no studies exploring the combined effects of these three movement behaviors. Future research should focus on integrated interventions that address PA, SB, and sleep to achieve a more comprehensive understanding of their collective impact on cognitive development in early childhood.

**Trial registration:**

This study was registered with PROSPERO under the registration number CRD42023479156.

**Supplementary Information:**

The online version contains supplementary material available at 10.1186/s12966-025-01705-y.

## Background

 Movement behaviors span a spectrum from sleep to vigorous physical activity (PA) [1]. Across a 24-hour period, individuals allocate varying amounts of time to PA, sedentary behavior (SB), and sleep. As time spent in one behavior increases, the time available for others decreases [2]. This creates a dynamic interaction that impacts overall health and development [3, 4]. Early childhood, particularly the preschool years (ages 3 to 5), is a critical period for developing key movement behaviors, including regular PA, adequate sleep, and limiting SB [5]. Establishing these behaviors during this period supports healthy growth and development [6]. In response to the growing recognition of these behaviors’ importance, various countries and global organizations like Canada and the World Health Organization (WHO) have introduced*“24-hour Movement Guidelines for the Early Years”* [7–12]. These guidelines provide recommendations for PA, SB, and sleep, and highlight the importance of movement behaviors in promoting overall health and well-being during early childhood.

The preschool years are an important period for brain plasticity, neurodevelopment, and the establishment of behavioral patterns [13]. During this time, children acquire essential skills that form the foundation for later academic achievement and overall well-being [14]. Cognitive competencies such as attention, memory, language, and executive function develop rapidly during this time and are influenced by numerous factors, including movement behaviors (i.e., PA, SB, and sleep) [15–19]. Movement behaviors established during this period are more likely to continue into later life, promoting lasting cognitive and health benefits. Early childhood, therefore, presents a unique opportunity to establish movement habits that may have enduring effects on cognitive development. This emphasizes the importance of understanding how movement behaviors contribute to cognitive development in early years.

Independently, PA has demonstrated significant benefits for neurocognitive development by enhancing brain plasticity, promoting synaptic growth, and supporting cognitive functions such as attention and executive functioning [20]. In contrast, excessive SB, particularly screen time, has been associated with cognitive deficits, including delayed language development and reduced executive functioning [21]. Adequate sleep also plays a crucial role in brain development, with higher sleep quality and sufficient duration contributing to improved memory consolidation, attention, and emotional regulation [22]. Despite evidence suggesting that PA may benefit cognitive domains in early childhood [23, 24], the role of PA in academic-related outcomes remains inconsistent due to variability in study designs and interventions [25]. Furthermore, evidence specific to SB and sleep in preschool-aged children is sparse, and the impact of interventions targeting these behaviors on cognitive outcomes is less understood. This highlights the need for further systematic reviews to better understand how interventions focused on individual movement behaviors affect cognitive development in young children, including the identification of optimal types, durations, and intensities.

Collectively, movement guidelines emphasize the integration of PA, SB, and sleep, recognizing that these behaviors interact dynamically and compete for time within a finite 24-hour period [26]. Since an increase in one behavior typically requires a decrease in another, examining these behaviors in isolation may yield incomplete conclusions [27, 28]. For instance, if an intervention successfully increases a child’s PA by 30 min, that time must be offset by reducing other movement behaviors. Increasing PA often reduces SB or alters sleep patterns, while inadequate sleep can negatively impact both cognitive functioning and the ability to engage in PA. Conversely, excessive SB may displace opportunities for PA or contribute to poor sleep quality. These behaviors are interdependent; changes in one can influence the others, collectively affecting physical health and cognitive outcomes [29, 30]. Therefore, adopting an integrated and balanced approach is critical to understanding how these movement behaviors interact and collectively impact health and cognition. However, evidence on the effects of combined movement behavior interventions on cognitive development in the early years remains limited.

Given that children’s daily behavioral experiences are crucial for their brain and cognitive development, understanding how PA, SB, and sleep independently and collectively influence cognition is critical [31]. Achieving recommendations for all three movement behaviors is vital for children’s health and development [32]. Numerous studies, to date, have examined the individual effects of movement behaviors on cognitive outcomes in preschoolers, but the findings are varied due to differences in study designs and methodologies. Randomized controlled trials (RCTs), known for their ability to minimize bias and establish causality, provide a reliable method for assessing these effects [33]. Additionally, while movement guidelines emphasize the interconnectedness of PA, SB, and sleep, few reviews have systematically explored these behaviors in an integrated manner, particularly in the context of preschool-aged children. Without this knowledge, it remains challenging to fully understand how these movement behaviors collectively influence cognitive development during this critical period. Therefore, this systematic review aims to critically evaluate the existing evidence from RCTs that target PA, SB, and sleep, both individually and in combination, to understand their effects on cognitive outcomes in early childhood. By synthesizing findings from high-quality studies, this review seeks to provide clearer guidance on the influence of movement behaviors on cognitive outcomes, identify optimal movement patterns that support cognitive development during this critical period, and inform evidence-based interventions tailored to this age group.

## Methods

### Study protocol and registration

This review was guided through the Preferred Reporting Items for Systematic Reviews and Meta-Analysis (PRISMA) 2020 statement for reporting systematic reviews and meta-analyses [34]. The search strategies were developed by two reviewers (NZ and CP), including databases, search terms, search date range, language restrictions, types of articles, and inclusion criteria. The strategies were constructed in consultation with a trained reference librarian and search specialist at the University of New Mexico Health Sciences Center. This review was registered with the International Prospective Register of Systematic Reviews (PROSPERO https://www.crd.york.ac.uk/prospero/; Registration no CRD42023479156).

## Search strategy

This review aimed to examine the impacts of movement behaviors (i.e., PA, SB, and sleep) on broader cognitive outcomes in preschool-aged children. The databases PubMed, PsycInfo, Web of Science, Embase, and CINAHL were used for the search, which was conducted between December 2023 and January 2024. The search terms were designed in line with the PICO strategy (Population – preschool children; Intervention – movement behaviors; Comparison – control group or normal activity; Outcome – cognition). To ensure comprehensive results, a combination of Medical Subject Headings (MeSH) and related keywords was employed. Where possible, database filters were applied to refine the results, although filter availability varied across platforms. PsycInfo, in particular, does not provide an easy filter for RCTs, so we utilized the search strategy recommended by Cochrane to address this limitation [35] (see Appendix A for a full description of the search strategy for each database). All retrieved studies were manually screened by two reviewers (CP and NZ) for relevance. Studies were first screened based on titles and abstracts, and potentially relevant full-text articles were then reviewed. References of relevant articles were also searched for additional results. The identified studies were stored using reference management software (EndNote 20, Thomson Reuters, New York, NY, USA).

## Inclusion criteria

Eligibility criteria required that studies (1) were published in English in peer-reviewed journals between January 2000 and December 2023; (2) employed a RCT design (individual or cluster-based); (3) included at least one movement behavior (PA, SB, or sleep), operationalized in alignment with the *Canadian 24-hour Movement Guidelines* [10]; (4) had a minimum intervention duration of four weeks, as research suggests this length is sufficient to produce positive mental and physical health outcomes [36]; (5) assessed cognition as a primary outcome. Cognition was broadly defined as a mental process by which an individual acquires knowledge or understanding, contributing to intellect, memory, or perception [24]. Broader aspects of cognition were reviewed, included but not limited to: academic achievement and/or performance, executive functions, learning, language, concentration/attention, memory, spatial processing, processing speed, emotion regulation, social cognition, creativity, and intelligence quotient (IQ), etc [37]. ; (6) included normatively developing preschool children, with an average sample age between 3 and 6 years old [24]; (7) utilized interventions that targeted the child directly, rather than focusing on caregivers; and (8) implemented interventions in the child’s home or school setting (e.g., childcare, early childhood education centers, Head Start programs, kindergarten, etc.).

## Synthesis

A pilot literature selection was conducted to ensure high inter-rater reliability among the reviewers. Following the inclusion and exclusion criteria, the lead author (CP) initially retrieved all relevant articles. A list of relevant articles was created in a Microsoft Excel spreadsheet (Microsoft Corporation, Redmond, WA, USA) and shared among all authors. All four authors independently completed two rounds of screening. In the first round, titles, abstracts, and keywords were reviewed for relevance after removing duplicates. In the second round, full-text screening was performed for articles deemed potentially eligible or with unclear eligibility. Each study was rigorously evaluated against pre-established criteria, and any conflicts were resolved through discussion until a consensus was reached. The selection process was documented in detail and summarized in a PRISMA flow diagram. Two independent reviewers (CP and NZ) extracted data from each article using a pre-specified form, which included: general information (publication date, first author, study location), participant characteristics (sample size, average age, sex), intervention setting; type; and dosage (duration, intensity, frequency), control conditions (e.g., waitlist, no intervention, or maintained routine/recess), cognitive outcomes (domains assessed), assessment tools, and main cognitive findings related to movement behaviors (see Table 1). Reviewers then compared extracted data and resolved any discrepancies through discussion until consensus was achieved.

**Table 1:** Study characteristics

Authors (year), country	Characteristics (*N*, age)	Intervention setting; type; and dosage (duration, intensity, frequency)	Control or Comparison	Cognition outcome	Cognition measurement tool	Main findings on cognition
iRCTs = 8						
Giordano & Alesi (2022), Italy [38]	*N* = 75 (39 boys; M_age_ = 5.68 ± 0.32 yrs) Intervention: *n* = 25 Control1: *n* = 25 Control2: *n* = 25	Setting; kindergarten Type; PA: Cognitively engaging PA (inhibiting previously learned movements, i.e., replacing walking on tiptoe with walking on heels) Dosage; 20 min/sessions, 3 sessions/week for 6 weeks	C1: Free play C2: maintained regular activity	Executive function (EF: motor and cognitive inhibition)	DNST; HSKT; GW; SD	Identified statistically significant differences between the two groups, with the IG showing greater improvement in DNST reaction time, HSKT accuracy, and GW reaction time
Hudson et al. (2021), USA [39]	*N* = 53 (*n* = 22 boys); M_age_ = 4.3 ± 0.6 yrs Intervention: *n* = 27 Control: *n* = 26	Setting; early childhood education center Type; PA: Gross motor skills (i.e., jumping) and fine motor skills Dosage; 30 min/session, 2 sessions/week for 8 weeks	Maintained regular activity	EF (inhibitory control, working memory, attention shifting, reaction time); early numeracy skills	EFT; Woodcock-Johnson IV: Applied problems subtest	Identified statistically significant differences between the two groups, with the IG showing greater improvement in overall EF, inhibitory control, and numeracy skills
Jarraya et al. (2019), Tunisia [40]	*N* = 45 (17 boys; M_age_ = 5.2 ± 0.4 yrs) Intervention1: *n* = 15 Intervention2: *n* = 15 Control: *n* = 15	Setting; kindergarten Type; PA1: Yoga PA2: Generic PE Dosage; 30 min/session, 2 sessions/week for 12 weeks	No PA	Visual attention (attention/EF, language, memory and learning, sensorimotor functions, and visuospatial processing) and Visual-motor precision (language, memory and learning, sensorimotor functions, and visuospatial processing, social perception, hyperactivity/impulsivity)	NEPSY-Visual Attention Test; NEPSY-II - Visuomotor Precision Test	Identified statistically significant differences between the IG and CG, with the Yoga showing greater improvement in visual attention, accompanied by a decrease in hyperactivity and inattention
Jarraya et al. (2022), Tunisia [41]	*N* = 52 (26 boys; M_age_ = 5.4 ± 0.2 yrs) Intervetnion1: *n* = 18 Intervetnion2: *n* = 17 Control: *n* = 17	Setting; kindergarten Type; PA1: Progressive muscle relaxation (PMR) PA2: Generic PE Dosage; 30 min/session, 2 sessions/week for 12 weeks	Free play	Attention and EF (memory, visuomotor precision, motor inhibition)	NEPSY-II - Visuomotor Precision Test; NEPSY-II - Statue Test; TBCT; RSFT	Identified statistically significant differences between the IG and CG, with the PMR showing greater improvement in attention time, attention score, visuomotor precision time error rates memorization, and motor inhibition
Liu et al. (2022), China [42]	*N* = 48 (23 boys; M_age_ = 4.90 ± 0.31 yrs) Intervention: *n* = 24 Control: *n* = 24	Setting; preschool Type; PA: Exergame dance movements Dosage; 30 min/session, 4 sessions/week for 4 weeks	Standard PE	EF (inhibition, working memory, shifting)	Early Years Toolbox (“Go/No-Go”; “Mr Ant”; “Card Sorting”)	Identified statistically significant differences between the two groups, with the IG showing greater improvement in all three EF tasks
Mulvey et al. (2018), USA [43]	*N* = 107 (51 boys; M_age_=5.14 *±* 0.81 yrs) Intervention: *n* = 50 Control: *n* = 57	Setting; preschool Type; PA: Successful Kinesthetic Instruction for Preschoolers (SKIP) program that aims to promote gross motor intervention (e.g., run, jump, leap, hop, gallop, slide, throw, catch, kick, dribble, strike, and roll) Dosage; 30 min/session, 2 sessions/week for 6 weeks	Maintained regular activity	EF (inhibitory control, working memory, and attentional focusing)	HTKS	Identified statistically significant differences between the two groups, with the IG showing greater improvement in EF
Shen et al. (2020), China [44]	*N* = 60 (30 boys; M_age_ = 4.37 ± 0.33 yrs) Intervention: *n* = 30 Control: *n* = 30	Setting; kindergarten Type; PA: Street-dance training Dosage; 40–50 min/session, 3 sessions/week for 8 weeks	Maintained regular activity	EF (response inhibition, executive attention, cognitive flexibility, working memory)	“Go/No-Go”; ANT; DCCS; BDS	Identified statistically significant differences between the two groups, with the IG showing greater improvement in working memory, inhibition control, and cognitive flexibility tasks
Wen et al. (2018), China [45]	*N* = 57 (31 boys; M_age_ = 4.4 ± 0.29 yrs) Intervention: *n* = 29 Control: *n* = 28	Setting; preschool Type; PA: Trampoline training program Dosage; 20 min/session, 5 sessions/week for 10 weeks	Maintained regular activity	EF (cognitive flexibility, inhibitory control, working memory)	“Go/No-Go”; SCA; FIS; WMS	No statistically significant differences were identified between the two groups
**cRCTs** **= 14**						
Alesi et al. (2021), Italy [46]	*N* = 174 (97 boys; M_age_ = 5.21 ± 0.45 yrs) Intervention: *n* = 110 Control: *n* = 64	Setting; kindergarten Type; PA: Gross motor skills (i.e., kicking) and fine motor skills ( i.e., copying shapes) Dosage; 60 min/session, 3 sessions/week for 10 weeks	Maintained regular school activities	Pre-literacy skills (linguistic understanding, oral expression, metacognition, cognitive abilities, reading/writing, math)	IPDA	Identified statistically significant differences between the two groups, with the IG showing greater improvement in linguistic comprehension, oral expression, metacognition, and other cognitive skills
Bai et al. (2022), China [47]	*N* = 62 (39 boys; M_age_ = 4.44 ± 0.46 yrs) Intervention: *n* = 30 Control: *n* = 32	Setting; preschool Type; PA: EF-focused exercise games: ‘Do the Opposite’ (opposite action), ‘Piggy Builds House’ (following instructions to build a house), ‘Flip the Cards’ (memory plus following motor instructions), soccer Dosage; 50 min/session, 3 sessions/week for 8 weeks	Maintained regular activity	EF (inhibitory control, working memory, shifting)	SSS; EH; DCCS	Identified statistically significant differences between the two groups, with the IG showing greater improvement in inhibitory control, working memory, and shifting
Burkhart et al. (2018), USA [48]	*N* = 71 (M_age_=3.8 ± 0.7 yrs) Intervention: *n* = 43 Control: *n* = 28	Setting; preschool Type; PA: Locomotor-based PA (running, hopping, skipping, galloping, sliding) Dosage; 30 min/session, 5 sessions/week for 24 weeks	Free play	Adaptive learning behaviors (hyperactivity, inattention); Inhibitory control	BASC-2; “Go/No-Go”	Identified statistically significant differences between the two groups, with the IG showing a greater decline in hyperactivity and a more pronounced improvement in attention
Ellis et al. (2019), Australia [49]	*N* = 115 (51 boys; 4.1 ± 0.7 yrs) Intervention: *n* = 55 Control: *n* = 60	Setting; childcare Type; SB: Reduce sitting time (height adjustable standing table/ easel; movement breaks, active story time, active mealtimes, standing when children do not want to nap) Dosage; 12 weeks	Maintained regular activity	EF (inhibition, working memory, shifting)	Early Years Toolbox (“Go/No-Go”; “Mr Ant”; “Card Sorting”)	No statistically significant differences were identified between the two groups
Mavilidi et al. (2015), Australia [50]	*N* = 111 (64 boys; M_age_*=*4.94 *±* 0.56 yrs) Intervention1: *n* = 31 Intervention2: *n* = 23 Control1: *n* = 31 Control2: *n* = 36	Setting; childcare Type; PA1: Integrated PA (actions indicated by the vocabulary word such as running and flapping hands for ‘fly’) PA2: Non-integrated PA (exercise unrelated to vocabulary word (such as running or walking regardless off vocabulary word) Dosage; 15 min/session, 2 sessions/week for 4 weeks	C1: Gesture (seated actions indicated by vocabulary word) C2: Conventional learning (verbally learning words without movement)	Memory performance (foreign language vocabulary learning)	Free recall test; Cued recall test	Identified statistically significant differences between the IG and CG, with the integrated PA showing greater improvement in free recall than all other groups, and in cued recall than CG groups
Mavilidi et al. (2017), Australia [51]	*N* = 90 (45 boys; M_age_ = 4.90 *±* 0.52 yrs) Intervention1: *n* = 30 Intervention2: *n* = 27 Control: *n* = 29	Setting; childcare Type; PA1: Integrated PA (ran from ‘planet to planet’, children learned the names of the planets and their order, based on the distance from the sun) PA2: Nonintegrated PA (running around room) Dosage; 10 min/session, one session/week for 4 weeks	Conventional learning: seated, observing planets during lesson	Science knowledge	Free recall test; Cued recall test	Identified statistically significant differences between the IG and CG, with the integrated PA showing greater improvement in learning outcomes
Mavilidi et al. (2018), Australia [52]	*N* = 120 (63 boys; M_age_ = 4.70 *±* 0.49 yrs) Intervention1: *n* = 30 Intervention2: *n* = 29 Control1: *n* = 29 Control2: *n* = 27	Setting; childcare Type; PA1: Integrated PA (ran, jumped, and stepped on numbers) PA2: Non-integrated PA (task unrelated PA) Dosage; 15 min/session, one session/week for 4 weeks	C1: Conventional sedentary teaching C2: Observing peers complete integrated PA while seated	Numeracy skills	Counting, number line estimation, block counting, numerical magnitude comparison, numerical identification	Identified statistically significant differences between the IG and CG, with the integrated PA showing greater improvement in math performance
Mavilidi et al. (2023), Australia [53]	*N* = 144 (79 boys; M_age_ = 4.41 *±* 0.61 yrs) Intervention1: *n* = 55 Intervention2: *n* = 48 Control: *n* = 41	Setting; childcare Type; PA1: Cognitively engaging PA (storytelling, cognitive activities, and motor tasks) PA2: Non-PA (storytelling and cognitive activities without motor tasks) Dosage; 15 min/session, two sessions/week for 6 weeks	Traditional storytelling	EF (inhibition, shifting, working memory); Self-regulation; Numeracy skills	Early Years Toolbox (“Go/No-Go”; “Mr Ant”; “Card Sorting”); SDQ; counting and numerical magnitude comparison tasks	No statistically significant differences were identified between the IG and CG
Miller et al. (2022), USA [54]	*N* = 116 (48 boys; M_age_ = 4.45 *±* 0.61 yrs) Intervention: *n* = 70 Control: *n* = 46	Setting; Head Start Type; PA: Children’s Health Activity Motor Program [CHAMP] Dosage; 3 days/week 16 weeks (48 sessions, 45 min/session, total dose = 2160 min)	Outdoor recess	Behavioral aspects of self-regulation; Cognitive aspects of self-regulation (working memory and cognitive flexibility)	HTKS; Early Years Toolbox (“Mr. Ant” and “Boats and Rabbits”)	Identified statistically significant differences between the two groups, with the IG showing greater improvement in behavioral aspects of self-regulation
Olive et al. (2023), Australia [55]	*N* = 314 (200 boys; M_age_=4.29 *±* 0.44 yrs) Intervention: *n* = 170 Control: *n* = 144	Setting; childcare Type; PA: Active Early Learning (AEL) program Dosage; 3–5 sessions of different components/week for 22 weeks	Maintained regular activity	EF (inhibition, working memory, attention shifting)	Early Years Toolbox (“Go/No-Go”; “Mr Ant”; “Card Sorting”)	Identified statistically significant differences between the two groups, with the IG showing greater improvement in inhibition
Piek et al. (2015), Australia [56]	*N*= 486 (257 boys; M_age_= 5.42 *±* 0.3 yrs) Intervention: =265 Control: *n* = 221	Setting; early childhood education center Type; PA: Animal Fun program (aims to enhance motor and social development) Dosage; 30 min/session, 4 sessions/week for 10 weeks	Maintained regular activity	Social-emotional development (hyperactivity and inattention)	SDQ	Identified statistically significant differences between the two groups, with the IG showing greater decrease in hyperactivity and inattention
Sanchez-Lopez et al. (2019), Spain [57]	*N* = 240 (105 boys; M_age_ = 5.84 ± 0.38 yrs) Intervention: *n* = 82 Control: *n* = 158	Setting; public and private school Type; PA: Multicomponent physical activity (PA) intervention (MOVI-KIDS) Dosage; 60 min/session, 3 sessions/week for one academic year	Standard PE	Cognitive performance (logical reasoning, verbal abilities, numerical abilities, spatial abilities, general intelligence)	BADyG E1	Identified statistically significant differences between the two groups, with the IG showing greater improvement in all cognitive variables
Schmidt et al. (2020), Switzerland [58]	*N* = 189 (98 boys; M_age_ = 5.34 ± 0.59 yrs) Intervention1: *n* = 75 Intervention2: *n* = 52 Control: *n* = 62	Setting; kindergarten Type; PA1: Physical-cognitive condition (Combination of EF-focused light-moderate PA) PA2: Sedentary-cognitive condition (EF-focused, low PA games) Dosage; 15 min/session, 4 sessions/week for 6 weeks	Maintained regular activity	EF (updating, inhibition, shifting)	N-back; DNST; DCCS	Identified statistically significant differences between the IG and CG, with the both IGs showing greater improvement in updating
Veiga et al. (2023), Portugal [59]	*N* = 233 (122 boys; M_age_ = 5.07 ± 0.84 yrs) Intervention: *n* = 155 Control: *n* = 78	Setting; preschool Type; PA: OUT to IN intervention (body-oriented) Dosage; 40 min/session, 20 sessions for 10 weeks	Maintained regular activity	Self-regulation	DNST; HTKS; SDQ	Identified statistically significant differences between the IG and CG, with the IG showing greater improvement in DNST and HTKS scores

*Abbreviation*:

iRCTs – individually randomized controlled trials

cRCTs – group/cluster randomized controlled trials

IG – intervention group

CG – control/comparison group

PA – physical activity

SB – sedentary behavior

*Tool – measure*

ADHD Rating Scale-IV – inattention, hyperactivity/impulsivity

Attention Network Test (ANT) – executive function

Backward Digit Span (BDS) task – working memory

Battery of General and Differential Aptitudes for schoolchildren (BADyG E1) – logical reasoning, verbal abilities, numerical abilities, spatial abilities, general intelligence

Behavior Assessment System for Children, 2nd edition (BASC-2) - behavioral, emotional, and adaptive functioning

Child Social Behavior Questionnaire – self regulation

Day-Night Stroop Test (DNST) –inhibition

Developmental Neuropsychological Assessment (NEPSY) Visual Attention Test – visual attention

Developmental Neuropsychological Assessment- 2nd Edition (NEPSY-II) Statue Test – motor persistence and inhibition

Developmental Neuropsychological Assessment- 2nd Edition (NEPSY-II) Visuomotor Precision Test – sensorimotor function/precision

Developmental Test of Visual-Motor Integration (VMI) - visual-motor integration

Dimensional Change Card Sort (DCCS) Test – cognitive flexibility, shifting

Early Years Toolbox “Card Sorting” – shifting

Early Years Toolbox “Go No-Go” – inhibition

Early Years Toolbox “Mr. Ant” – visual-spatial working memory

Emotion Vocabulary Questionnaire (EVQ) - emotion communication

Empty House (EH) task – working memory

Executive Function Touch (EFT) – inhibitory control, working memory, attention shifting

Flexible Item Selection (FIS) task – cognitive flexibility

Gift Wrap (GW) – delay of gratification/inhibitory control

Head-Shoulders-Knees-Toes test (HSKT) – executive function; motor inhibition

Heads Toes, Knees Skip (HTKS) - executive function

Intelligence and development scales (IDS) - spatial working memory

N-back task – updating

Questionario per l’Identificazione Precoce delle Difficoltà di Apprendimento (IPDA) - pre-literacy skills

Rey Simple Figure Test (RSFT) – visuo-constructional abilities and visual memory

Silly Sound Stroop (SSS) task – inhibitory control

Snack Delay (SD) – ability to inhibit pre-potent or automatic responses

Spatial Conflict Arrow (SCA) task – inhibitory control

Strengths and Difficulties Questionnaire (SDQ) - hyperactivity/inattention

Teddy Bear Cancellation Test (TBCT) – attention and executive functioning

Woodcock-Johnson IV Applied problems subtests – quantitative ability

Working Memory Span (WMS) - working memory

## Effect size calculations

It is important to note that relying solely on p-values to interpret research findings has limitations, as *p*-values indicate statistical significance but do not convey the magnitude or practical importance of an effect. Moreover, p-values are sensitive to sample size, which can lead to potentially misleading conclusions, such as overestimating significance in larger samples or underestimating it in smaller ones [60]. To address this, we calculated effect sizes for interventions with statistically significant results, including all significant cognitive outcomes identified in the studies.

Effect sizes provide a standardized measure of the magnitude of the effect, independent of sample size [61]. This approach enables better quantification and comparison of the effects across studies, a clearer understanding of intervention impacts, and facilitates the practical interpretation and application of findings. Specifically, we computed Cohen’s d to interpret effect size. Data on pre-test and post-test means and standard deviations were extracted from the results sections and were calculated using the formula: [d = (Meanpost - Meanpre)/SDpooled], where the pooled standard deviation (SD) was computed as: [SDpooled = sqrt((SDpre^2 + SDpost^2)/2)] [62]. Cohen’s d was interpreted as small (0.2), medium (0.5), or large (0.8) [62].

For studies reporting follow-up assessments, only post-test scores were used for effect size calculations to avoid the influence of time-related factors and ensure a consistent measure of immediate intervention impacts. All calculations were performed using RStudio (version 2024.04.2) (RStudio, PBC, Boston, MA, http://www.rstudio.com/).

### Risk of bias

The Revised Cochrane risk-of-bias tool for randomized trials (RoB 2) [63] and the Revised Cochrane risk-of-bias tool for cluster-randomized trials (RoB 2 CRT) [64] were used to assess the quality of individually randomized parallel-group trials and cluster-randomized parallel-group trials, respectively. These tools evaluate each study based on multiple criteria, including the randomization process, adherence to the intended intervention, allocation concealment, blinding (during both the study and outcome assessment phases), completeness of outcome data, selective reporting, and other potential sources of bias. Each study was assigned an overall risk-of-bias rating of “low,” “high,” or “unclear.” All authors independently assessed the quality of the studies, and any disagreements were resolved through discussion until consensus was reached. The interrater reliability was measured using Cohen’s kappa, which resulted in a score of 0.78, indicating substantial agreement among the reviewers.

## Results

### Search results

**Fig. 1:**
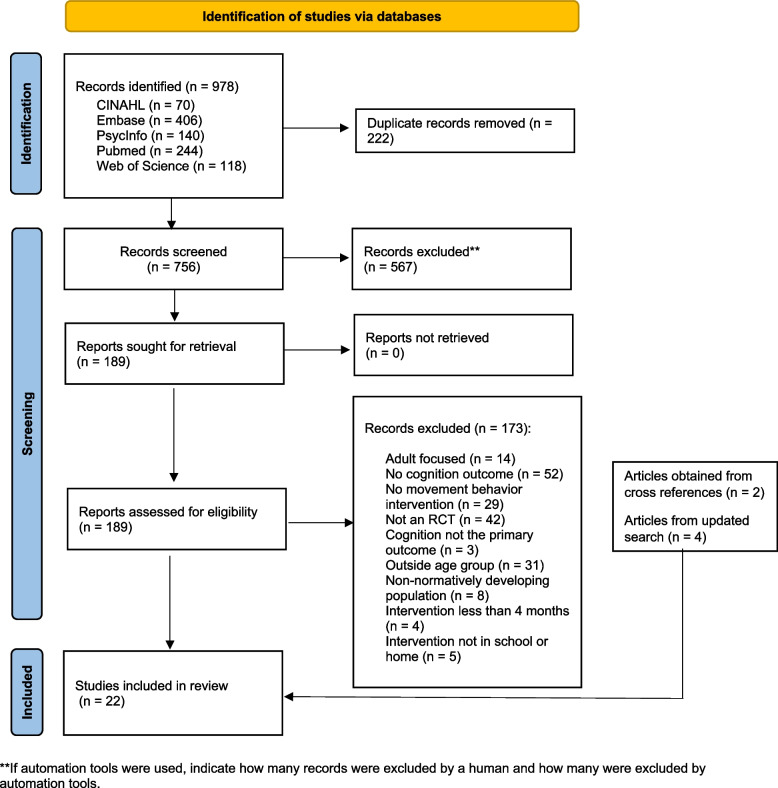
PRISMA flow diagram of report selection

The initial search yielded 978 results. After removing duplicates, 756 records were screened based on titles and abstracts. Of these, 566 articles were excluded for not meeting relevance criteria, leaving 190 reports for full-text retrieval. After further excluding 169 articles that did not meet the inclusion criteria, 21 studies remained from the initial search. An additional study was identified through reference list searches, bringing the total number of included studies to 22. Of these, 14 were individual RCTs, and 8 were cluster RCTs (Fig. 1).

### Study characteristics

The studies included in this review were published between 2015 and 2023, representing a wide geographical range. These studies were conducted in eight countries: Italy (2 studies) [38, 46], USA (4 studies) [39, 43, 48, 54], Tunisia (2 studies) [40, 41], China (4 studies) [42, 44, 45, 47], Australia (7 studies) [49–53, 55, 56], Spain (1 study) [57], Switzerland (1 study) [58], and Portugal (1 study) [59]. Participants’ ages ranged from approximately 3.8 to 5.84 years, with sample sizes varying between 45 and 486 children. The proportion of boys across studies ranged from 40 to 60%, reflecting balanced gender representation. The diversity in participant characteristics and geographical distribution enhances the generalizability of the findings across different cultural and demographic contexts.

### Intervention characteristics

All included studies were conducted in structured school-based environments such as early childcare centers, kindergartens, or preschools, ensuring consistency in intervention implementation and participant engagement. Across the 22 studies, a wide variety of intervention types, durations, frequencies, and session lengths were documented. PA was the predominant focus (21 studies), with only one study targeting SB [49] and none exclusively addressing sleep or multi-behavior interventions. PA interventions ranged from gross motor activities (e.g., jumping, running, and dance) to cognitively engaging tasks designed to improve executive function (e.g., “Simon Says”-type games or yoga).

The average intervention duration was approximately 10.1 weeks, with the shortest lasting 4 weeks and the longest spanning a full academic year. Session frequencies varied from 1 to 5 times per week, with most studies implementing 2 to 3 sessions per week. The duration of each session ranged from 10 to 60 min, with an average session length of approximately 30 min (average = 28.68 min).

Control or comparison groups primarily maintained regular activity (11 studies), participated in free play (2 studies), or attended standard PE classes (2 studies). Other controls included no PA (1 study), outdoor recess (1 study), and traditional sedentary classroom teaching (2 studies). Certain studies utilized multiple control groups, such as a combination of free play and maintained regular activities.

### Outcomes of interest and their assessments

The cognitive outcomes assessed across the studies varied widely, with inconsistent measures utilized to evaluate similar constructs. Executive function was the most frequently assessed domain and is widely understood to encompass core components such as inhibition, working memory, and shifting. For example, the Early Years Toolbox “Go/No-Go” task was frequently used to measure inhibition, while the Dimensional Change Card Sort (DCCS) test assessed cognitive flexibility and shifting. Some tools, such as the Head-Toes-Knees-Shoulders (HTKS) test, are used to assess multiple executive function components and behavioral aspects of self-regulation simultaneously. In this review, we adopted a broad framework for executive function to capture the diversity of approaches used in the included studies, while acknowledging that heterogeneity in its operationalization could complicate comparisons.

Other cognitive outcomes assessed included numeracy skills (e.g., counting) and language and literacy (e.g., linguistic comprehension, pre-literacy skills, and cognitive abilities related to reading and writing). Memory outcomes included free recall and cued recall, while attention outcomes, such as visual attention, attention span, and reaction time, were assessed separately from executive function. Social-emotional development outcomes, such as hyperactivity and inattention, were also measured independently and were not categorized under executive function in most studies.

These variations in outcome classification and measurement highlight the methodological heterogeneity across the studies, reflecting differences in conceptual frameworks and tools used to assess cognitive domains. This methodological diversity, particularly in the assessment of executive function, emphasizes the need for standardized frameworks in future research. Although it is theoretically possible to conduct a meta-analysis on the cognitive outcomes reported, the substantial heterogeneity in assessment methods introduces significant biases and limits the validity and reliability of any meta-analytic conclusions. Consequently, neither a meta-analysis nor a subgroup analysis was feasible for this review.

### Intervention effects

Of the 22 included studies, 19 reported statistically significant improvements in cognitive outcomes following PA interventions, while three found no significant effects. Table 2 displays the effect sizes for these 19 effective interventions, showing that PA interventions integrating cognitive engagement, such as executive function-focused games and integrated physical activities with learning components, produced the largest effect sizes (Cohen’s d > 0.8). For example, Giordano & Alesi (2022) observed a large effect size in inhibition tasks (Cohen’s d = 2.33 for the Day-Night Stroop Task) following a cognitively engaging PA intervention. This program involved replacing previously learned movements, such as substituting walking on tiptoes with walking on heels, in 20-minute sessions three times per week over six weeks [38]. Bai et al. (2022) utilized executive function-focused exercise games, such as “Do the Opposite” and “Flip the Cards,” delivered in 50-minute sessions three times per week for eight weeks. This intervention yielded large effect sizes for inhibitory control, working memory, and shifting tasks [47]. This intervention yielded large effect sizes in inhibitory control, working memory, and shifting tasks. Additionally, Mavilidi et al. (2018) employed integrated physical activities where children engaged in running, jumping, and stepping on numbers in a game-like setting. Conducted in 15-minute sessions once per week for four weeks, the intervention demonstrated significant improvements in numeracy skills and cognitive performance (Cohen’s d > 0.8) [52]. These findings highlight the advantages of integrating cognitive and physical components into intervention programs to achieve meaningful cognitive gains.

**Table 2:** Effect size calculations for significant cognitive outcomes across all interventions

Reference	Outcome	Intervention	Pre-Test Mean	Post-Test Mean	Change in Mean (ΔM)	Pooled SD	Effect Size (Cohen’s d)
Giordano & Alesi (2022) [38]	Reaction Time (DNST)	PA	−1.56	0.75	−2.32	0.996	−2.33
	Accuracy (HSKT)		9.20	17.00	7.80	5.52	1.41
	Reaction Time (GW)		0.61	0.75	0.14	0.38	0.37
Hudson et al. (2021) [39]	Overall EF	PA	59.3	67.2	7.9	14.67	0.54
	Inhibitory Control		57.1	69.1	12.0	22.56	0.53
	Numeracy Skills		94.1	96.6	2.5	17.31	0.14
Jarraya et al. (2019) [40]	Visual Attention (A)	Yoga	1.41	1.31	−0.10	0.061	−1.27
		PE	1.41	1.35	−0.06	0.055	−0.66
	Visual Attention (B)	Yoga	8.87	12.20	3.33	1.63	1.50
		PE	8.93	10.53	1.60	1.17	0.54
	Hyperactivity	Yoga	13.93	9.60	−4.33	1.63	−1.71
		PE	13.67	11.27	−2.40	1.44	−0.66
	Inattention	Yoga	12.40	6.87	−5.53	1.57	−2.61
		PE	12.73	10.27	−2.46	1.28	−1.25
Jarraya et al. (2022) [41]	Attention (A)	PRM	2.9	1.6	−1.3	0.73	−1.78
		PE	2.9	2.1	−0.8	0.65	−1.23
	Attention (B)	PRM	10.3	12.4	2.1	1.85	1.14
		PE	10.4	10.9	0.5	1.70	0.29
	Visuomotor Precision Time	PRM	24.9	21.4	−3.5	1.80	−1.94
		PE	24.2	23.3	−0.9	1.60	−0.56
	Error Rates (Visuomotor)	PRM	11.2	8.6	−2.6	1.45	−1.79
		PE	11.6	10.8	−0.8	1.40	−0.57
	Memorization	PRM	18.2	23.1	4.9	1.76	2.78
		PE	18.4	20.3	1.9	2.30	0.83
	Motor Inhibition	PRM	1.8	4.6	2.8	1.63	1.72
		PE	1.6	3.1	1.5	2.00	0.75
Mulvey et al. (2018), USA [43]	Executive Function (HTKS)	SKIP	12.31	19.08	6.77	10.76	0.63
Liu et al. (2022) [42]	Inhibition	PA	0.71	0.84	0.13	0.14	0.95
	Shifting		5.13	6.92	1.79	2.56	0.70
	Working Memory		1.71	2.53	0.82	0.72	1.14
Shen et al. (2020) [44]	Working Memory	PA	30.97	34.53	3.56	5.04	0.71
	Inhibition Control		28.80	33.33	4.53	5.18	0.87
	Cognitive Flexibility		1.77	3.73	1.96	2.35	0.84
Alesi et al. (2021) [46]	Linguistic Comprehension	PA	9.45	11.25	1.80	1.61	1.12
	Oral Expression		14.22	17.37	3.15	3.13	1.00
	Metacognition		11.39	14.15	2.76	2.34	1.18
	Other Cognitive Skills		30.34	36.62	6.28	5.11	1.23
Bai et al. (2022) [47]	Inhibition Control (Behavioral Performance)	PA	16.87	19.43	2.56	2.47	1.04
	Working Memory (Behavioral Performance)		10.43	13.47	3.04	2.07	1.47
	Shifting (Behavioral Performance)		15.47	18.10	2.63	2.13	1.24
	Inhibition Control (Reaction Time)		320.75	301.55	−19.20	20.34	−0.94
	Working Memory (Reaction Time)		290.45	275.62	−14.83	19.49	−0.76
	Shifting (Reaction Time)		305.25	288.47	−16.78	18.44	−0.91
Burkart et al. (2018) [48]	Hyperactivity	PA	14.53	11.95	−2.58	3.07	−0.84
	Attention		15.02	13.43	−1.59	3.68	−0.43
Mavilidi et al. (2015) [50]	Free Recall	Integrated PA	0.98	2.63	1.65	1.40	1.18
	Cued Recall		1.81	5.61	3.80	2.20	1.73
Mavilidi et al. (2017) [51]	Learning Outcomes	Integrated PA	1.58	15.53	13.95	3.67	3.80
Mavilidi et al. (2018) [52]	Match Performance	Integrated PA	27.72	39.08	11.36	12.54	0.91
Miller et al. (2022), USA [54]	Behavioral Self-Regulation (HTKS)	CHAMP	20.4	33.1	12.7	7.77	1.63
Olive et al. (2023) [55]	Inhibition	AEL	0.61	0.72	0.11	0.20	0.54
Piek et al. (2015) [56]	Hyperactivity/Inattention	Animal Fun	8.34	6.12	−2.22	2.17	−1.02
Sanchez-Lopez et al. (2019) [57]	Logical Reasoning	MOVI-KIDS	25.34	36.51	11.17	8.38	1.14
	Verbal Factor		22.36	27.69	5.33	4.26	1.17
	Numerical Factor		14.15	26.09	11.94	8.97	0.99
	Spatial Factor		13.72	20.13	6.41	7.28	1.09
	General Intelligence		50.24	73.92	23.68	15.38	1.48
Schmidt et al. (2020) [58]	Updating (Accuracy)	Physical-cognitive condition	15.41	17.49	2.08	3.12	0.67
		Sedentary-cognitive condition	14.25	17.33	3.08	3.14	0.98
Veiga et al. (2023) [59]	Self-Regulation (DNST)	OUT to IN	0.61	0.91	0.30	0.28	1.07
	Self-Regulation (HTKS)		12.71	33.75	21.04	16.81	1.25

Moderate effect sizes (Cohen’s d = 0.5–0.8) were observed in interventions emphasizing gross motor activities, progressive muscle relaxation, and exergame-based PA. For instance, Liu et al. (2022) reported moderate improvements in working memory (Cohen’s d = 0.7) and attention-shifting tasks (Cohen’s d = 0.5) through exergame dance movements delivered four times per week for four weeks [42]. Similarly, Hudson et al. (2021) achieved moderate effects in numeracy skills (Cohen’s d = 0.54) and inhibitory control (Cohen’s d = 0.53) following an intervention combining gross and fine motor skill activities conducted twice weekly over eight weeks [39]. Jarraya et al. (2022) also demonstrated moderate improvements in visuomotor precision (Cohen’s d = 0.54) using progressive muscle relaxation sessions delivered twice per week for 12 weeks [41]. These studies illustrate the effectiveness of moderate-intensity PA interventions that combine PA with cognitive elements in improving specific cognitive outcomes.

Interventions with small effect sizes (Cohen’s d < 0.5) generally focused on narrow domains such as linguistic comprehension, specific numeracy tasks, and inhibitory control. For example, Olive et al. (2023) implemented the Active Early Learning program, consisting of 3–5 sessions per week over 22 weeks, which produced small improvements in inhibition tasks (Cohen’s d = 0.54) [55]. Likewise, Shen et al. (2020) employed street-dance training involving 40–50-minute sessions three times per week over eight weeks, achieving small effect sizes in cognitive flexibility tasks (Cohen’s d = 0.4) [44]. While these interventions demonstrated limited impact, they offered incremental cognitive benefits, particularly in specialized areas such as working memory and cognitive shifting. The modest effects observed may stem from limited intervention intensity or frequency, such as 6-week programs with two 20-minute sessions per week, which may not provide sufficient exposure for robust cognitive changes. Additionally, interventions targeting single domains, such as motor inhibition or cognitive flexibility, may lack the holistic stimulation required for broader cognitive development. The choice of activities, like street-dance training, might not fully align with children’s interests or cognitive needs, reducing intrinsic motivation and engagement. These findings underscore the importance of tailoring interventions to children’s developmental and cognitive profiles, increasing their intensity and duration, and ensuring that activities align with targeted cognitive outcomes for greater effectiveness.

Effect sizes, overall, calculated for significant cognitive outcomes, highlighted the pronounced impact of cognitively engaging PA interventions on executive function domains, including inhibition, working memory, and cognitive flexibility in preschool-aged children. However, the lack of evidence on SB and sleep interventions highlights critical gaps in understanding the broader role of 24-hour movement behaviors in cognitive development.

### RoB Assessment

**Fig. 2:**
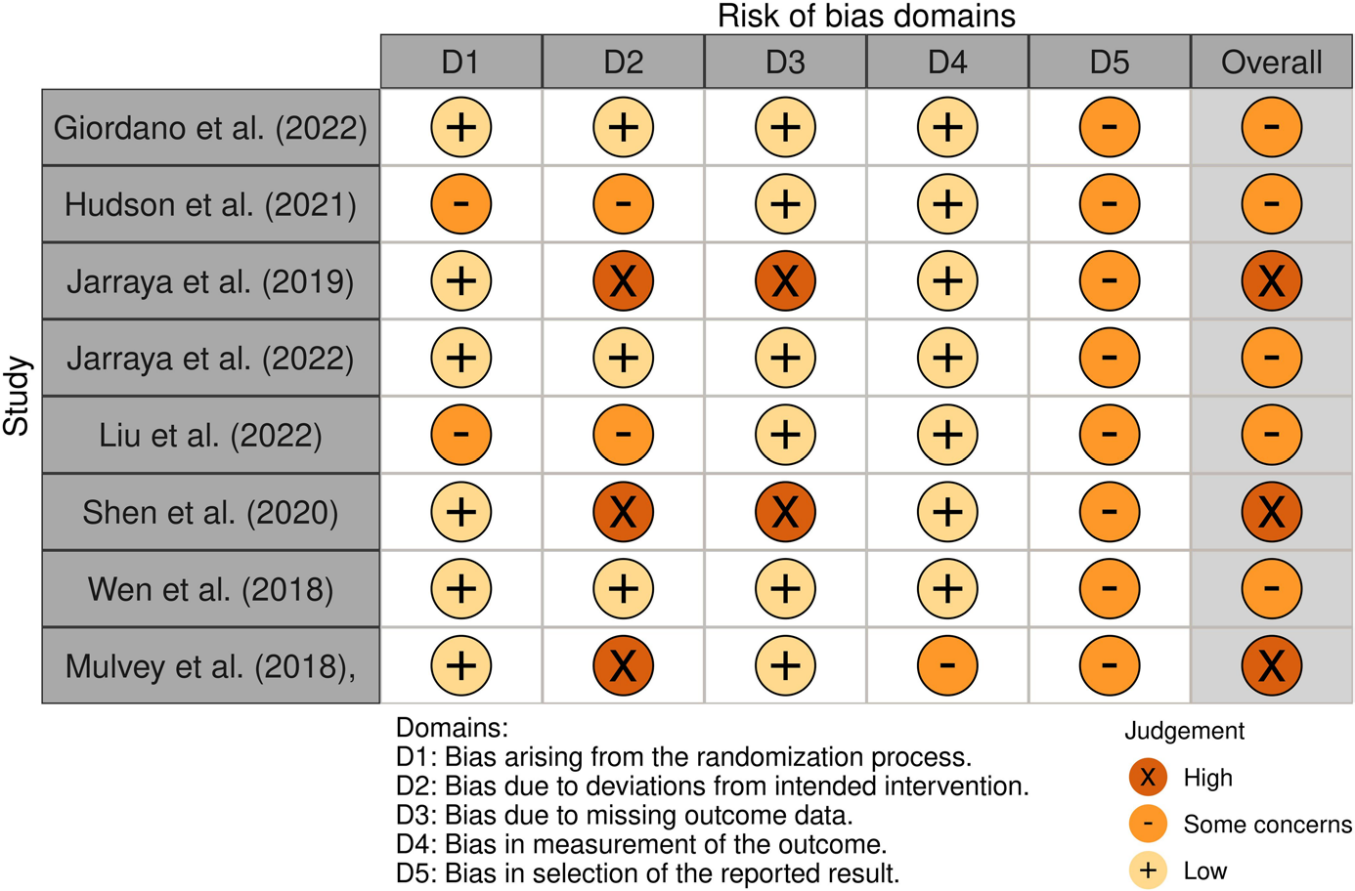
ROB2-individual

**Fig. 3:**
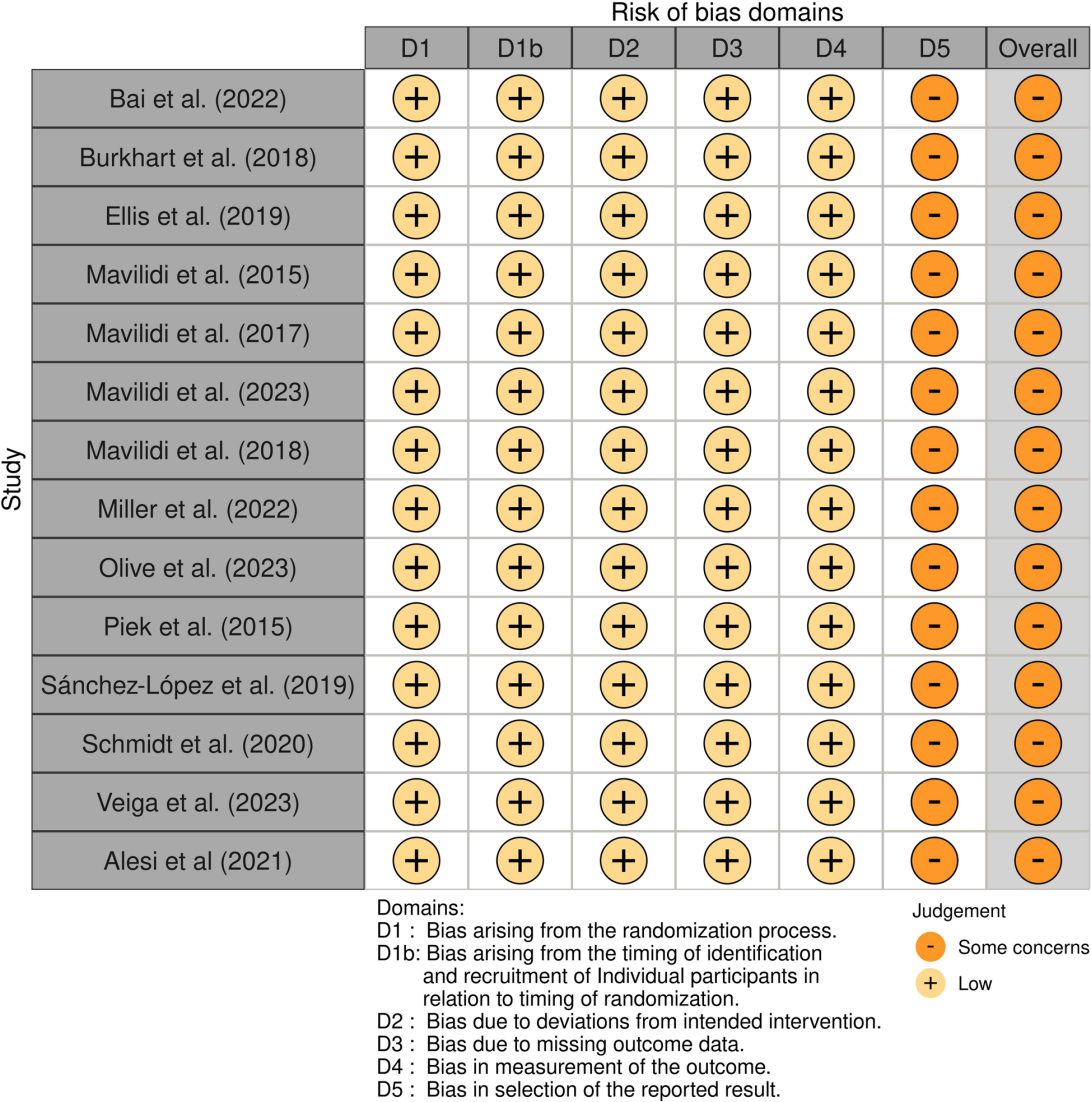
ROB2-Cluster

**Fig. 4:**
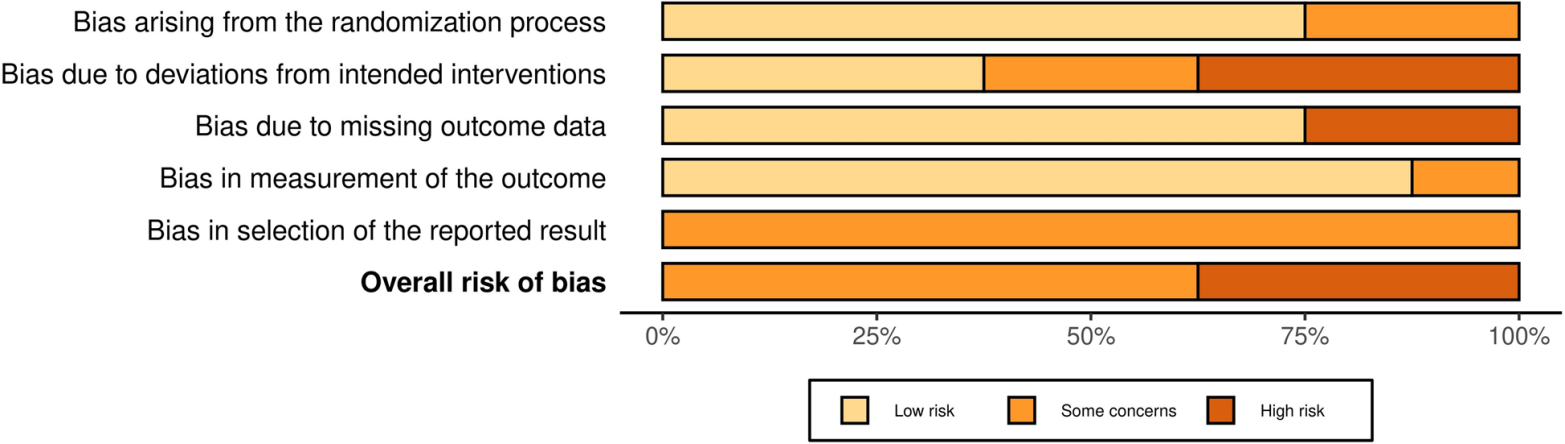
ROB2-overall

 The overall quality of the 14 individual RCTs was low, with five studies rated as having “some concerns” and three rated as having “high” RoB. None were rated as having “low” RoB. Similarly, the quality of the eight cluster RCTs was also low, with all studies rated as having “some concerns.” It is important to note that the Cochrane RoB tool is highly rigorous, making it particularly challenging for non-clinical behavioral interventions (where subjective influence is difficult to control) to achieve a “low” risk rating. For detailed assessments of each study and the overall RoB across different domains, see Figs. 2 and 3, and 4.

## Discussion

The *24-Hour Movement Guidelines* emphasize the importance of balancing PA, SB (including screen time), and sleep throughout the day. The combination of these movement behaviors plays a critical role in shaping overall health in young children [10]. The preschool years are a critical period for cognitive development, and there is growing interest in identifying effective interventions to enhance cognitive skills during this time. This review synthesizes evidence from recent RCTs that examine the effects of interventions targeting movement behaviors, either individually or in combination, on various aspects of cognitive development in early childhood. We included 14 individual RCTs and 8 cluster RCTs and observed that movement behaviors, particularly PA interventions with a cognitively engaging element, have a positive impact on various aspects of cognition in preschoolers. However, the effects of interventions targeting SB, sleep, or combinations of these behaviors on cognition were less conclusive due to the limited number of studies in these areas.

Our findings are consistent with previous reviews, which suggest that PA interventions have a positive effect on cognitive development in early childhood, while also highlighting subtle differences across study designs and outcomes. For instance, both Carson et al. (2016) [23] and Zeng et al. (2017) [24] demonstrate that PA interventions consistently yield statistically significant benefits for cognitive outcomes, particularly in executive function, attention, and language, which aligns with the results of our review. However, Carson’s review included both observational and experimental studies, offering a broader perspective on PA’s influence on cognition. In contrast, our review focuses exclusively on RCTs, providing stronger causal evidence of PA’s beneficial effects on cognitive development in young children. Additionally, while Zeng’s review included fewer RCTs and did not explore the dose-response relationship between PA and cognition, our review addresses this gap by providing a more detailed analysis of intervention characteristics, such as duration, frequency, and intensity. Moreover, Li et al. (2020) [36] conducted a meta-analysis demonstrating that chronic PA interventions positively influenced executive functions, including inhibition and working memory, in young children, which aligns with the findings of our review. Notably, Li’s study highlighted that the type of PA (e.g., PA-only vs. PA combined with cognitive challenges) moderated the effects on executive functions. Our review expands on this by emphasizing the enhanced benefits of cognitively engaging PA activities over and above simply being physically active, which were shown to improve outcomes in certain trials. This suggests that integrating cognitive tasks within PA may yield stronger effects on cognitive development compared to PA alone. Similarly, the most recent meta-analysis by Morales et al. (2024) [65] found that PA interventions positively impacted various cognitive outcomes, including attention, inhibition, and working memory, further supporting our findings [66]. Interestingly, Morales’s review also noted that interventions lasting longer than three weeks were most effective, reinforcing our assumption that longer intervention durations might lead to more substantial cognitive benefits, as our review included only interventions lasting more than four weeks.

Our review identified a significant underrepresentation of interventions targeting SB, which limits the conclusiveness of findings in this area. The single study that focused on SB reported no significant cognitive improvements, suggesting that reducing sedentary time alone may not be sufficient to enhance cognitive outcomes in young children [49]. One explanation for the lack of significant cognitive improvements may be the passive nature of SB reduction strategies. Standing tables and increased activity during mealtimes may not be sufficiently stimulating for cognitive development compared to more interactive and physically engaging interventions. In contrast, interventions incorporating both physical and cognitive challenges, such as those involving active play or structured exercise programs, have been shown to produce more substantial cognitive gains in children than passive strategies [67–69]. This suggests that simply reducing sedentary time without incorporating stimulating activities may fall short of promoting cognitive improvements. This finding aligns with prior research indicating that cognitive benefits in young children may require more comprehensive and engaging approaches rather than simply reducing SB. For instance, a meta-analysis by Carson et al. (2015) found that while increased PA was consistently linked to better cognitive performance in children, interventions focusing solely on reducing SB were less effective in producing cognitive benefits [21]. Therefore, future interventions to limit SB may benefit from incorporating more engaging activities, possibly integrating cognitive challenges to enhance cognitive benefits.

Emerging evidence highlights the critical role of sleep in cognitive development, particularly in processes such as memory consolidation, synaptic pruning, and emotional regulation [70]. Sleep facilitates the transfer of information from short-term to long-term memory, a process essential for learning and knowledge retention [66, 71]. Adequate sleep duration and quality are further associated with improved attention, executive functioning, and emotional stability [72]. In early childhood, these cognitive functions are foundational, underpinning the acquisition of language, self-regulation, problem-solving skills, and social interactions [73, 74]. Despite such evidence, sleep interventions for preschool-aged children remain underexplored, with no RCTs specifically targeting sleep outcomes in the context of cognitive development identified in our review. This absence represents a significant gap, as studies involving older children have shown that sleep hygiene interventions can enhance both academic performance and behavioral outcomes [75]. Therefore, investigating the effects of structured sleep interventions such as establishing consistent bedtime routines, reducing screen time before sleep, and maintaining a regular sleep schedule, on the cognitive development of preschoolers could provide invaluable insights.

We failed to locate any RCTs that examined the effects of combined movement behaviors on cognitive outcomes in young children. Current literature suggests that interventions addressing multiple aspects of 24-hour movement behaviors may offer comprehensive and synergistic benefits for cognitive health. For instance, a meta-analysis by Song et al. (2022) demonstrated that integrating interventions focused on both sleep and PA can yield greater cognitive benefits in children compared to targeting a single behavior alone [76]. This aligns with a growing body of evidence suggesting that these behaviors are not independent but interact in complex ways that affect brain development and function. A recent review by Wang et al. (2024) also highlighted the potential for combined interventions across 24-hour movement behaviors to positively influence mental health and cognitive performance in children and adolescents [77]. The review underscored the importance of considering the interplay between these behaviors, advocating for more RCTs that simultaneously address PA, SB, and sleep. Such studies could more accurately reflect the holistic nature of how movement behaviors contribute to cognitive development in young children, particularly in a real-world context where these behaviors are interrelated. Although research in this area is still emerging, early evidence supports the benefits of a multi-behavior approach. For example, a recent parent-focused RCT targeting all three 24-hour movement behaviors in preschool children found that incorporating sleep, PA, and SB management led to improvements in behavioral outcomes [78]. While the study focused on behavioral health, it did not include direct measures of cognitive outcomes. This leaves a gap in understanding how combined 24-hour movement behaviors might affect cognitive function in early childhood. The lack of RCTs investigating the cognitive outcomes of multi-behavior interventions points to an urgent need for future research. By designing studies that address all aspects of 24-hour movement behaviors, researchers could discover new ways to support cognitive development during early childhood, a critical time when the brain is highly adaptable. With the known benefits of sleep and PA for brain health, and growing evidence of the harms of too much SB, there is a strong reason to explore these connections further.

### Strengths and limitations

A key strength of this review is its exclusive focus on RCTs, which are considered the gold standard for establishing causal relationships between interventions and outcomes. Additionally, focusing exclusively on studies with cognition as a primary outcome strengthens the validity of our findings by eliminating ambiguity and ensuring that cognitive improvements were the central objective of the included interventions. Furthermore, we comprehensively reviewed all movement behaviors and their combinations, despite most studies focusing primarily on PA interventions. To our knowledge, this is the first systematic review investigating the causal relationship between movement behaviors and cognitive outcomes in young children. We also calculated effect sizes (Cohen’s d) for interventions with statistically significant results, providing a standardized measure of intervention impact independent of sample size. This approach facilitates better comparisons across studies and offers clearer insights into the efficacy of interventions on cognitive outcomes. However, several limitations should be noted. First, the conceptualization of cognition in this review may not have captured all relevant outcomes. Cognition is a broad and loosely defined construct, making it challenging to establish a universally accepted definition or include every potential keyword in the search. Second, the heterogeneity in intervention types, durations, cognitive measures, and assessment tools across studies complicates efforts to synthesize the findings through meta-analysis or sub-analyses, which limits our ability to quantify overall effect sizes. To address these challenges, future research should prioritize standardized assessment tools and harmonized outcome measures. Consistent measurement approaches will enhance the comparability of findings across studies, enabling more meaningful subgroup analyses and more robust evidence synthesis. Another notable limitation of this review is the RoB, with many studies rated as having “some concerns” or “high risk.” Factors such as lack of blinding and issues with allocation concealment contribute to the overall low quality of the included studies. This limitation emphasizes the need for cautious interpretation and call for future trials to implement standardized, blinded methodologies to minimize bias and enhance reliability. As studies reporting significant or positive findings are more likely to be published and included in systematic reviews, publication bias could be a potential limitation as well. While we conducted a comprehensive search to include studies with null or non-significant results, the possibility of bias in the available evidence cannot be entirely ruled out. Last but not least, our search may not be fully comprehensive, as it was limited to several key databases. Relevant databases such as SPORTDiscus were not included, which may have resulted in the exclusion of some potential articles on the topic. This limitation highlights the possibility of missing studies that could have contributed to the findings of this review.

## Conclusions

This review demonstrates that PA interventions significantly enhance cognitive outcomes in early childhood, particularly in executive functions such as inhibition, attention, and cognitive flexibility. Interventions incorporating cognitively engaging activities showed the largest effects, while motor skill-focused and general PA programs yielded moderate to smaller gains. Specific cognitive skills, such as linguistic comprehension and numeracy, showed incremental improvements, highlighting the variability of outcomes based on intervention type and design. These findings highlight the importance of early implementation of tailored PA interventions to support cognitive development in young children. Optimizing outcomes requires careful consideration of the type, intensity, and frequency of interventions. However, further research is needed to investigate the effects of SB, sleep, and combinations of movement behaviors, offering a more comprehensive understanding of their collective role in early cognitive development.

## Supplementary Information

Supplementary Material 1.

Supplementary Material 2.

## Data Availability

No datasets were generated or analysed during the current study.

## Abbreviations

CINAHL: Cumulative Index to Nursing and Allied Health Literature
DCCS: Dimensional Change Card Sort
HTKS: Head-Toes-Knees-Shoulders
IQ: Intelligence Quotient
MeSH: Medical Subject Headings
PA: Physical Activity
PICO: Population, Intervention, Comparison, Outcome
PRISMA: Preferred Reporting Items for Systematic Reviews and Meta-Analyses
RCT: Randomized Controlled Trial
RoB: Risk of Bias
SB: Sedentary Behavior
SD: Standard Deviation
SEM: Standard Error of the Mean
WHO: World Health Organization
