# Supplementary material for: Effects of movement behaviors on preschoolers’ cognition: a systematic review of randomized controlled trials
Source: Int J Behav Nutr Phys Act. 2025 Jan 23;22:12. doi: 10.1186/s12966-025-01705-y (PMC11755889; doi:10.1186/s12966-025-01705-y)
Supplement: Supplementary file 1 — Supplementary Material 1. [file 12966_2025_1705_MOESM1_ESM.docx]

**Appendix A. Search Strategy**

| **Database** |  | **Search Terms** | **Filters Applied** | **Number of results** |
| --- | --- | --- | --- | --- |
| CINAHL |  | ("Sedentary Behavior" OR "Life Style" OR "Exercise Movement Technique" OR "Exercise" OR "Physical Fitness" OR "Physical Endurance" OR "Psychomotor Performance" OR "Screen Time" OR "screen time" OR "motor skill" OR "cardiovascular fitness" OR “movement behavior” OR “sedentary behavior” OR “fitness” OR “physical activity” OR “sleep”) AND (“Cognition" OR "Comprehension" OR "Executive Function" OR "Attention" OR "Problem Solving" OR "Discrimination Learning" OR "Verbal Learning" OR "Thinking” OR "Academic Performance" OR "cognitive development" OR "school readiness" OR “academic performance” OR “vocabulary” OR “cognitive ability”) | English; peer reviewed; research article; preschool (2-5); human; randomized controlled trial; after 1999 | 70 |
| Embase |  | ("Sedentary Behavior" OR "Life Style" OR "Exercise Movement Technique" OR "Exercise" OR "Physical Fitness" OR "Physical Endurance" OR "Psychomotor Performance" OR "Screen Time" OR "screen time" OR "motor skill" OR "cardiovascular fitness" OR “movement behavior” OR “sedentary behavior” OR “fitness” OR “physical activity” OR “sleep”) AND (“Cognition" OR "Comprehension" OR "Executive Function" OR "Attention" OR "Problem Solving" OR "Discrimination Learning" OR "Verbal Learning" OR "Thinking” OR "Academic Performance" OR "cognitive development" OR "school readiness" OR “academic performance” OR “vocabulary” OR “cognitive ability”) | English; article; preschool (1-6); human; randomized controlled trial; 2000-2023 | 406 |
| PsycInfo |  | ("Sedentary Behavior" OR "Life Style" OR "Exercise Movement Technique" OR "Exercise" OR "Physical Fitness" OR "Physical Endurance" OR "Psychomotor Performance" OR "Screen Time" OR "screen time" OR "motor skill" OR "cardiovascular fitness" OR “movement behavior” OR “sedentary behavior” OR “fitness” OR “physical activity” OR “sleep”) AND (“Cognition" OR "Comprehension" OR "Executive Function" OR "Attention" OR "Problem Solving" OR "Discrimination Learning" OR "Verbal Learning" OR "Thinking” OR "Academic Performance" OR "cognitive development" OR "school readiness" OR “academic performance” OR “vocabulary” OR “cognitive ability”) AND (SU.EXACT("Treatment Effectiveness Evaluation") OR SU.EXACT.EXPLODE("Treatment Outcomes") OR SU.EXACT("Placebo") OR SU.EXACT("Followup Studies") OR placebo* OR random* OR "comparative stud*" OR clinical NEAR/3 trial* OR research NEAR/3 design OR evaluat* NEAR/3 stud* OR prospectiv* NEAR/3 stud* OR (singl* OR doubl* OR trebl* OR tripl*) NEAR/3 (blind* OR mask*)) | English; human; scholarly journal; preschool (2-5); peer reviewed (scholarly journals); exclude dissertations; 2000-2023 | 140 |
| Pubmed |  | ("Sedentary Behavior*"[Mesh] OR "Life Style"[Mesh] OR "Exercise Movement Technique*"[Mesh] OR "Exercise"[Mesh] OR "Physical Fitness"[Mesh] OR "Physical Endurance" OR "Psychomotor Performance"[Mesh] OR "Screen Time"[Mesh] OR "screen time" OR "motor skill*" OR "cardiovascular fitness" OR “movement behavior” OR “sedentary behavior” OR “fitness” OR “physical activity” OR “sleep”) AND (“Cognition"[Mesh:NoExp] OR "Comprehension"[Mesh] OR "Executive Function"[Mesh] OR "Attention"[Mesh] OR "Problem Solving"[Mesh] OR "Discrimination Learning"[Mesh] OR "Verbal Learning"[Mesh] OR "Thinking"[Mesh:NoExp] OR "Academic Performance"[Mesh] OR "cognit*" OR "cognitive development" OR "school readiness" OR “academic performance” OR “vocabulary” OR “cognitive ability”) AND ("Child, Preschool"[Mesh] OR "preschool” OR "young child*" OR “early years”) | Human; Randomized controlled trial; from 2000 | 244 |
| Web of Science |  | ("Sedentary Behavior" OR "Life Style" OR "Exercise Movement Technique" OR "Exercise" OR "Physical Fitness" OR "Physical Endurance" OR "Psychomotor Performance" OR "Screen Time" OR "screen time" OR "motor skill" OR "cardiovascular fitness" OR “movement behavior” OR “sedentary behavior” OR “fitness” OR “physical activity” OR “sleep”) AND (“Cognition" OR "Comprehension" OR "Executive Function" OR "Attention" OR "Problem Solving" OR "Discrimination Learning" OR "Verbal Learning" OR "Thinking” OR "Academic Performance" OR "cognitive development" OR "school readiness" OR “academic performance” OR “vocabulary” OR “cognitive ability”) AND (randomised controlled trial or randomized controlled trial or rct or randomized clinical trial or randomised clinical trial or experimental design) AND ("preschool" OR "young children" OR "young child" OR "early years") | Date between 1/1/2000 – 12/31/2023; article | 118 |
